# Risk of sharing resistant bacteria and/or resistance elements between dogs and their owners

**DOI:** 10.1186/s12917-022-03298-1

**Published:** 2022-05-27

**Authors:** Zahra Naziri, Meisam Poormaleknia, Azar Ghaedi Oliyaei

**Affiliations:** Department of Pathobiology, School of Veterinary Medicine, Shiraz University, Shiraz, Iran

**Keywords:** *Escherichia coli*, Extended spectrum beta-lactamase, Integrons, Antibiotic-resistance, Dog, Dog-owner

## Abstract

**Background:**

The indiscriminate use and the similarity of prescribed antibiotics especially beta-lactams in human and small animal medicine, along with the close communication between pets and humans, increases the risk of the transfer of antibiotic-resistant bacteria and/or resistance elements especially integrons, between them. Therefore, we aimed to compare the frequencies of extended spectrum beta-lactamase (ESBL)-producing strains, major ESBL genes, classes 1 and 2 integrons, and antibiotic resistance patterns of fecal *Escherichia coli* (*E. coli*) isolates from dogs and their owners.

**Methods:**

The present study was conducted on 144 commensal *E. coli* isolates from the feces of 28 healthy dog-owner pairs and 16 healthy humans who did not own pets. Phenotypic confirmatory test was used to identify the frequencies of ESBL-producing *E. coli*. Frequencies of *bla*_CTX-M_, *bla*_SHV_, and *bla*_TEM_ genes, and also classes 1 and 2 integrons were determined by polymerase chain reaction. Resistance against 16 conventional antibiotics was determined by disk diffusion technique.

**Results:**

ESBL-production status was similar between the *E. coli* isolates of 71.4% of dog-owner pairs. The *E. coli* isolates of 75, 60.7, and 85.7% of dog-owner pairs were similar in terms of the presence or absence of *bla*_CTX-M_, *bla*_TEM_, and *bla*_SHV_ genes, respectively. The presence or absence of class 1 and class 2 integrons was the same in *E. coli* isolates of 57.1% of dog-owner pairs. Prevalence of resistance to chloramphenicol and tetracycline was significantly higher in *E. coli* isolates of dogs than owners, but for other 10 (83.3%) tested antibiotics, no statistically significant difference was found in prevalence of antibiotic resistance between dogs and owners isolates. Furthermore, the antibiotic-resistance profile was the same in the *E. coli* isolates of 14.3% of dog-owner pairs.

**Conclusions:**

The results of current research highlight the seriousness of the drug-resistance problem and the need to prevent further increases and spread of antibiotic-resistance to reduce treatment failure. Moreover, relatively similar characteristics of the *E. coli* isolates of dogs and their owners can show the risk of sharing resistant bacteria and/or resistance elements between them.

## Background

In the last decades, the indiscriminate use of antibiotics in human and veterinary medicine imposed selection pressure on bacteria, particularly the microbiota. Moreover, the similarity of prescribed antibiotics in human and small animal medicine, along with the close communication between companion animals and humans, increased the risk of the transfer of antibiotic-resistant bacteria between pets and humans [1, 2].

Integrons are one of the genetic elements that have a role in the horizontal transmission of antibiotic resistance genes [3]. Gut microbiota consists of various bacterial species which accumulate in close vicinity. Therefore, multi-resistance integrons in the microbiota can have a major role in preserving and spreading antibiotic resistance, as well as the development of multi-drug resistance (MDR) phenotypes among pathogenic and commensal bacteria [4].

Beta-lactam antibiotics are broad-spectrum drugs widely prescribed for treating infections in humans and animals. Resistance to these medications is mainly mediated by the production of beta-lactamase enzymes by bacteria [5]. The rising global rates of extended-spectrum beta-lactamase (ESBL) producing bacteria, particularly *Escherichia coli* (*E. coli*) and also methicillin-resistant staphylococci (MRS), such as methicillin-resistant *Staphylococcus aureus* (MRSA) among humans and pets may cause the inefficacy of treatment with such important medicines [6]. In addition, the concurrency of ESBL production and resistance to other antibiotic classes can further limit the therapeutic options for bacterial infections [7].

The spread of antibiotic resistance genes and resistant bacteria among humans and companion animals is of public health importance, and microbiota has a significant role in this process. Therefore, we aimed to determine and compare the frequencies of ESBL-producing strains, major ESBL genes including *bla*_CTX-M_, *bla*_SHV_, and *bla*_TEM_, classes 1 and 2 integrons, and antibiotic resistance patterns among commensal *E. coli* isolates in the feces of healthy dogs and their owners in Shiraz, Iran. Furthermore, the associations between ESBL production, the presence of major ESBL genes, the presence of class 1 and class 2 integrons, and resistance to some conventional antibiotics were investigated in this study.

## Results

### Phenotypic identification of ESBL-producing *E. coli*

The prevalence of ESBL-producing *E. coli* among all 144 *E. coli* isolates and in each studied group is separately reported in (Table 1). The results of statistical analysis showed no significant difference in the prevalence of ESBL-producing *E. coli* between the isolates of dogs and owners (*P* = 0.188), between dog and control human isolates (*P* = 0.705), and also between owner and control human isolates (*P* = 0.122).

**Table 1:** Prevalence of ESBL-producing, *bla*_CTX-M_, *bla*_SHV_, and *bla*_TEM_-positive, class 1 and class 2 integrons-positive, MDR, and antibiotic-resistant *E. coli* among the studied isolates^a^

Characteristics	In all isolates (***n*** = 144)	In dog’s isolates (***n*** = 56)	In owner’s isolates (***n*** = 56)	In control’s isolates (***n*** = 32)
**ESBL-producing**	19 (13.2)	6 (10.7)	11 (19.6)	2 (6.2)
***bla***_**CTX-M**_**-positive**	21 (14.6)	7 (12.5)	12 (21.4)	2 (6.2)
***bla***_**SHV**_***-*positive**	7 (4.9)	4 (7.1)	1 (1.8)	2 (6.2)
***bla***_**TEM**_**-positive**	61 (42.4)	24 (42.9)	25 (44.6)	12 (37.5)
**Class 1 integron-positive**	52 (36.1)	23 (41.1)	22 (39.3)	7 (21.9)
**Class 2 integron-positive**	16 (11.1)	4 (7.1)	7 (12.5)	5 (15.6)
**MDR**	42 (29.2)	23 (41.1)	13 (23.2)	6 (18.8)
**Cephalexin-resistant**	19 (13.2)	5 (8.9)	12 (21.4)	2 (6.2)
**Cefoxitin-resistant**	1 (0.7)	0 (0.0)	1 (1.8)	0 (0.0)
**Ceftazidime-resistant**	29 (20.1)	12 (21.4)	15 (26.8)	2 (6.2)
**Cefotaxime-resistant**	46 (31.9)	20 (35.7)	22 (39.3)	4 (12.5)
**Cefepime-resistant**	19 (13.2)	8 (14.3)	9 (16.1)	2 (6.2)
**Aztreonam-resistant**	9 (6.2)	4 (7.1)	5 (8.9)	0 (0.0)
**Amikacin-resistant**	7 (4.9)	3 (5.4)	4 (7.1)	0 (0.0)
**Streptomycin-resistant**	60 (41.7)	27 (48.2)	21 (37.5)	12 (37.5)
**Norfloxacin-resistant**	5 (3.5)	5 (8.9)	0 (0.0)	0 (0.0)
**Nalidixic acid-resistant**	37 (25.7)	8 (14.3)	14 (25.0)	15 (46.9)
**Chloramphenicol-resistant**	20 (13.9)	13 (23.2)	3 (5.4)	4 (12.5)
**Tetracycline-resistant**	56 (38.9)	30 (53.6)	17 (30.4)	9 (28.1)

^a^Values are shown as number (%)

Among 28 dog-owner pairs, *E. coli* isolates of 20 (71.4%) pairs were similar in terms of ESBL-production as *E. coli* isolates of 2 (7.1%) and 18 (64.3%) dog-owner pairs were ESBL-producer and not ESBL-producer, respectively.

### Detection of major ESBL genes in *E. coli* isolates

The prevalence of major ESBL genes-harboring *E. coli* among all 144 *E. coli* isolates, and in each studied group is separately reported in (Table 1). Among the three studied ESBL genes, the *bla*_TEM_ gene had the highest prevalence followed by *bla*_CTX-M_ and *bla*_SHV_ in all *E. coli* isolates and the *E. coli* isolates of dogs and owners separately. The prevalence of *bla*_TEM_, *bla*_CTX-M_, and *bla*_SHV_ genes in these isolates was significantly different (*P* < 0.001). However, *bla*_CTX-M_ and *bla*_SHV_ genes had a similar prevalence (*P* = 1) in control human *E. coli* isolates, significantly lower than the prevalence of the *bla*_TEM_ gene (*P* < 0.001) (Table 1). The results of statistical analysis showed no significant difference in the prevalence of ESBL genes between dogs and owners isolates, between dogs and control human isolates, and also between owners and control human isolates (*P* > 0.05).

Overall, none of the isolates had the three ESBL genes simultaneously. Seventy (48.6%) isolates comprising 28 (50.0%) dog isolates, 24 (42.8%) owner isolates, and 18 (56.2%) control human isolates were negative for all the three tested ESBL genes. The statistical analysis revealed no significant association between the simultaneous presences of ESBL genes (*P* > 0.05).

**Fig. 1:**
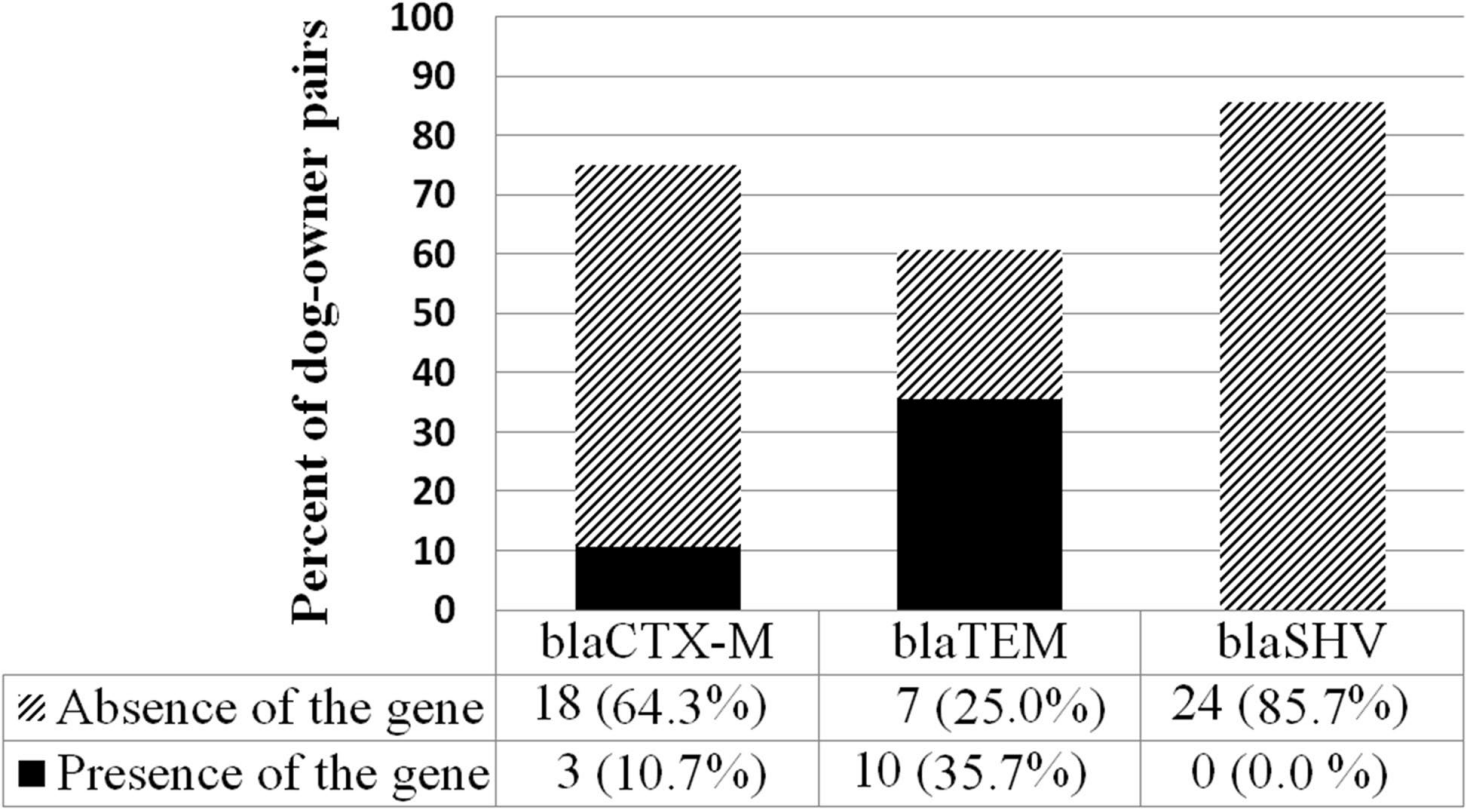
Proportion of dog-owner pairs who had *E. coli* isolates with a similar status of presence or absence of ESBL genes

Out of 28 dog–owner pairs, *E. coli* isolates of 8 (28.6%) pairs had the same patterns of the presence of ESBL genes, and *E. coli* isolates of 5 (17.9%) pairs had none of the tested ESBL genes. *E. coli* isolates of 24 (85.7%), 21 (75.0%), and 17 (60.7%) dog-owner pairs had the similar status of presence or absence of *bla*_SHV_, *bla*_CTX-M_, and *bla*_TEM_ genes, respectively. The proportion of dog-owner pairs who had *E. coli* isolates with a similar status of presence or absence of ESBL genes are demonstrated in (Fig. 1).

### Detection of class 1 and class 2 integrons in *E. coli* isolates

The prevalence of class 1 integron- and class 2 integron-positive *E. coli* among all 144 *E. coli* isolates, and in each of the studied groups are separately reported in (Table 1). We found that the prevalence of class 1 integrons was significantly higher than class 2 integrons in all 144 *E. coli* isolates and each of the three studied groups (*P* < 0.001).

Among all 144 *E. coli* isolates, 15 (10.4%) had both class 1 and class 2 integrons, 37 (25.7%) only had class 1 integrons, 1 (0.7%) only had class 2 integrons, and 91 (63.2%) were negative for both class 1 and class 2 integrons.

The statistical analysis indicated a significant association between the presence of class 1 integron and class 2 integron (*P* < 0.001). As a result, 93.8% of *E. coli* isolates which had class 2 integron also had class 1 integron simultaneously.

Out of 28 dog-owner pairs, *E. coli* isolates of 8 (28.6%) pairs had similar integron classes, and in 8 (28.6%) pairs, the isolates of dogs and their owners were negative for both classes 1 and 2 integrons.

Overall, the results of statistical analysis showed no significant difference in the prevalence of class 1 and class 2 integrons between dogs and owners isolates, between dogs and control human isolates, and between owners and control human isolates (*P* > 0.05).

### Antibiotic resistance patterns of *E. coli* isolates

The prevalence of antibiotic-resistant *E. coli* in all 144 *E. coli* isolates and each of the studied groups is separately presented in (Table 1). Noteworthy, all the control human isolates were susceptible to cefoxitin, amikacin, aztreonam, and norfloxacin. The frequency of resistance to cephalexin, ceftazidime, and cefepime was below 10.0% in these isolates. In dogs, all isolates were susceptible to cefoxitin and the frequency of resistance to amikacin, aztreonam, cephalexin, and norfloxacin was below 10.0%. In owners, all isolates were susceptible to norfloxacin and the frequency of resistance to cefoxitin, chloramphenicol, amikacin, and aztreonam was below 10.0% (Table 1).

We observed that the prevalence of resistance to chloramphenicol (*P* = 0.007) and tetracycline (*P* = 0.013) was significantly higher in *E. coli* isolates of dogs than in owners. However, no significant difference was found in the prevalence of antibiotic resistance between dogs and owners isolates for the other tested antibiotics (*P* > 0.05). Furthermore, the prevalence of resistance to cefotaxime (*P* = 0.019) and tetracycline (*P* = 0.021) in dogs isolates was significantly higher than in control human isolates. In contrast, the prevalence of resistance to nalidixic acid was significantly higher in *E. coli* isolates of control humans than dogs (*P* = 0.001). No significant difference was found in the prevalence of antibiotic resistance between dogs and control human isolates for the other tested antibiotics (*P* > 0.05).

Comparison of the results of antibiotic resistance in *E. coli* isolates of owners and control humans showed that resistance to ceftazidime (*P* = 0.019) and cefotaxime (*P* = 0.008) was significantly higher in isolates of owners than in control humans. On the other hand, resistance to nalidixic acid was significantly higher in *E. coli* isolates of control humans than in owners (*P* = 0.036). Regarding other tested antibiotics, no significant difference was found in the prevalence of antibiotic resistance between owners and control humans isolates (*P* > 0.05).

Resistance or susceptibility to cefoxitin, amikacin, norfloxacin, cephalexin, cefepime, aztreonam, chloramphenicol, nalidixic acid, tetracycline, ceftazidime, cefotaxime, and streptomycin was similar in 96.2, 85.7, 82.1, 78.6, 78.6, 78.6, 67.9, 60.7, 60.7, 57.1, 50.0, and 42.9% of the dog-owner pairs, respectively. In four (14.3%) dog-owner pairs, *E. coli* isolates of dogs and their owners had the same antibiotic resistance profiles.

Among 144 *E. coli* isolates, 42 (29.2%) were resistant to at least one antimicrobial agent in three or more classes of antimicrobial drugs, being considered MDR isolates [8]. The prevalence of MDR *E. coli* isolates in dogs was significantly higher than in owners (*P* = 0.043) and control humans (*P* = 0.032). Although the prevalence of MDR *E. coli* isolates in owners (23.2%) was higher than in control humans (18.8%), this difference was not statistically significant (*P* = 0.624).

### Associations of ESBL production, presence of major ESBL genes, presence of class 1 and class 2 integrons, and resistance to antibiotics

Overall, the results of statistical analysis showed a significant association between ESBL production and resistance to cephalexin, ceftazidime, cefotaxime, cefepime, and aztreonam (*P* < 0.001). Moreover, resistance to the above antibiotics was significantly associated with the *bla*_CTX-M_ gene (*P* < 0.001). The presence of the *bla*_TEM_ gene was significantly associated with resistance to streptomycin (*P* = 0.024), chloramphenicol (*P* = 0.007), and tetracycline (*P* = 0.004). However, no significant association was found between the presence of the *bla*_SHV_ gene and resistance to any of the tested antibiotics (*P* > 0.05).

Among 19 ESBL -producing *E. coli* isolates, 18 (94.7%) were positive for at least one of the tested ESBL encoding genes. In addition, *bla*_CTX-M_, *bla*_TEM_, and *bla*_SHV_ genes were present in 78.9, 57.9, and 0.0% of the ESBL producer isolates, respectively. However, only the presence of the *bla*_CTX-M_ gene was significantly associated with the ESBL production ability of *E. coli* isolates (*P* < 0.001).

Investigation of the association between integrons and resistance to antibiotics revealed that among the tested antibiotics, only resistance to chloramphenicol (*P* = 0.004) and tetracycline (*P* = 0.040) was significantly associated with class 1 integron.

The results of statistical analysis showed no significant association between the presence of class 1 and class 2 integrons and ESBL production and also the presence of the tested ESBL genes in *E. coli* isolates (*P* > 0.05).

## Discussion

In the present study, ESBL production status was similar between the *E. coli* isolates of 71.4% of dog-owner pairs. Moreover, the *E. coli* isolates of 75, 60.7, and 85.7% of dog-owner pairs were similar in terms of the presence or absence of *bla*_CTX-M_, *bla*_TEM_, and *bla*_SHV_ genes, respectively. The presence or absence of class 1 and class 2 integrons was the same in *E. coli* isolates of 57.1% of dog-owner pairs. Furthermore, we observed a high similarity in resistance or susceptibility to 12 tested antibiotics in the *E. coli* isolates of dog-owner pairs, along with the absence of a significant difference in the prevalence of resistance to 83.3% of tested antibiotics between dogs and owners isolates. The antibiotic-resistance profile was the same in the *E. coli* isolates of 14.3% of dog-owner pairs. All the mentioned findings may indicate the possibility of transmitting resistant bacteria and/or resistance elements between dogs and their owners.

The possibility of the circulation of antibiotic-resistant *E. coli* clones or the direct/ indirect transmission of resistance elements such as plasmids or integrons between humans and pets in the same environment has been reported in previous studies [9–11]. For instance, in the study by Harada et al. (2012) relatively similar characteristics, including phylogenetic groups, antibiotic resistance, and virulence profiles, were observed in fecal *E. coli* isolated from dogs and their owners [11]. Likewise, in an investigation by Stenske et al. [10], no significant difference was found in the prevalence of resistance to 17 antibiotics between dogs and owners *E. coli* isolates. Carvalho et al. [9] also revealed similar resistance patterns, ESBL genes, and ESBL production ability in *E. coli* isolates from dogs and their owners in the same households. Ljungquist et al. [12] reported that dogs with owners carrying extended-spectrum cephalosporin-resistant *Enterobacteriaceae* (ESCRE) strains had identical ESCRE strains. On the other hand, when owners did not carry ESCRE strains, these strains were not found in dogs. According to these results, they reported the transfer of ESCRE strains between humans and dogs. These finding are in agreement with ours.

In the present study, the prevalence of MDR *E. coli* isolates in dogs was significantly higher than in owners and control humans. Likewise, several studies indicated the importance of MDR *E. coli* strains in pets and their effect on the treatment of in contact human infections [9, 13]. Moreover, Carvalho et al. [9] suggested that dogs can be a source of MDR *E. coli* in a household. Similarly, in a study by Abbas et al. [13], the prevalence of ESBL-producing *E. coli* (81.8% in dogs vs. 59% in owners) and multidrug-resistant ESBL-producing *E. coli* (44.4% in dogs vs. 30.77% in owners) was higher in dogs than owners. In line with the previous investigations, in our study, the frequency of resistance to cefotaxime and tetracycline was higher in dogs isolates, and resistance to ceftazidime and cefotaxime was higher in owners isolates in comparison with control humans isolates. Exclusively resistance to nalidixic acid was higher in control humans isolates than in dogs and owners isolates. These findings can indicate the importance of dogs and close contact with them in spreading antibiotic-resistant *E. coli* strains.

The prevalence of antibiotic-resistant and MDR *E. coli* isolates was variable in dogs and owners in the previous studies [9–11, 13]. Overall, the different prevalence of antibiotic resistance and MDR between the *E. coli* isolates of our study and previous reports could be due to variations in the origins of the isolates, type and amount of antibiotics used in humans and pets of different countries and therefore differences in the selective pressures in the *E. coli* strains of diverse geographical areas, and variation in the type and extent of communication between dogs and their owners in distinct cultures. In Iran, as an Islamic country, the extent of communication between dogs and their owners may be somewhat less than in other countries. For example some behaviors might be less frequent, such as allowing dogs to lick the owners’ faces. Moreover, Iranian dog owners usually wash their hands after petting dogs and before eating food. In addition, the higher level of antibiotic resistance in the *E. coli* isolates of the present study could be attributed to the free access and arbitrary use of antibiotics in Iran, which is a major problem for both medicine and veterinary medicine.

In the current research, associations were found between ESBL production, the presence of the *bla* gene, and resistance to beta-lactam antibiotics. In addition, the presence of the *bla*_TEM_ gene was significantly associated with resistance to streptomycin, chloramphenicol, and tetracycline. These findings agree with those reported by Demirel et al. [7], who declared therapeutic complications caused by the simultaneous presence of ESBL genes and other classes of antibiotic-resistance genes including aminoglycosides, chloramphenicol, tetracyclines, fluoroquinolones and trimethoprim-sulfamethoxazole resistance genes in the identical plasmids.

Sáenz et al. [14] found various resistance genes in commensal *E. coli* strains from humans and animals. They stated that normal flora could play a major role in transmitting antibiotic-resistance elements, especially integrons. In the present study, no significant difference was found in the prevalence of class 1 and class 2 integrons between dogs and owners isolates, between dogs and control human isolates, and between owners and control human isolates. These results are consistent with those observed by Skurnik et al. [15]. The latter researcher reported that the distribution of gene cassettes in the integrons of pet *E. coli* isolates was very similar to those of human commensal *E. coli* isolates.

In line with our study, the prevalence of class 1 integrons was significantly higher than class 2 integrons in the previous studies [16–18]. Furthermore, the prevalence of class 1 and class 2 integrons was almost similar in these studies and our research. However, in a study by Akya et al. [19], the prevalence (92.3%) of class 1 integrons was much more than in our study. This inconsistency can be due to variations in the origin and type of studied strains, as their study was on ESBL-positive *E. coli* strains which were isolated from humans with urinary tract infections.

Investigation of the association between class 1 and class 2 integrons and resistance to antibiotics revealed a significant association between the presence of class 1 integrons and resistance to chloramphenicol and tetracycline. As well, Akya et al. [19] found a significant association between the presence of class 1 integrons and resistance to ceftazidime, streptomycin, and trimethoprim-sulfamethoxazole. Moreover, Kheiri & Akhtari [18] reported a significant association between *dfr* (dihydrofolate reductase) and *aad* (aminoglycoside adenyltransferase) gene cassettes in the integrons and resistance to trimethoprim-sulfamethoxazole and streptomycin, respectively. These findings can demonstrate the role of integrons in the preservation and transmission of antibiotic resistance genes.

Overall, relatively similar characteristics such as antimicrobial-resistance profiles and the presence or absence of ESBL genes and integrons in the *E. coli* isolates of dogs and their owners can indicate the possibility of the transmission of resistant bacteria and/or resistance elements between dogs and their owners. However, further studies such as pulsed-field gel electrophoresis (PFGE) or core genome multi-locus sequence typing (MLST), are needed to confirm that the strains are identical. In our previous study, the genetic relatedness of *E. coli* isolates from dogs and their owners was investigated by PFGE, enterobacterial repetitive intergenic consensus-polymerase chain reaction (ERIC-PCR), and randomly amplified polymorphic DNA (RAPD) analyses [20].

## Conclusion

The results of the current research highlight the seriousness of the drug-resistance problem and the need to prevent further increases and spread of antibiotic-resistance to reduce treatment failure. Moreover, relatively similar characteristics of the *E. coli* isolates of dogs and their owners can show the risk of sharing resistant bacteria and/or resistance elements between them.

## Methods

### Bacterial isolates

The present study was conducted on a total of 144 *E. coli* isolates from the feces of 28 healthy dog-owner pairs and 16 healthy humans who did not own the pet animals, as a control group. Two microbiologically confirmed *E. coli* isolates from each of the stool samples previously collected in a study by Naziri et al. [21] were included in this study. Briefly, the stool samples were previously streaked on Eosin Methylene Blue agar (Merck, Germany) and were incubated at 44 °C for 24 hours. Two random colonies with metallic green sheen morphology were selected from each sample. Gram-staining, oxidase, catalase, and IMViC (indole, motility, Voges-Proskauer, and citrate) tests were applied to these selected colonies to confirm the *E. coli* isolates.

All human participants were over 18 years old and filled out the informed consent. Dogs and dog owners were selected from the clients of several veterinary clinics in Shiraz, Iran and also the clients of the small animal clinics of the School of Veterinary Medicine, Shiraz University.

### Phenotypic identification of ESBL-producing *E. coli*

The clinical and laboratory standards institute (CLSI) phenotypic confirmatory test was used to identify ESBL-producing *E. coli* [22]. At least five millimeters rise in the zone of inhibition around the cefotaxime-clavulanic acid or ceftazidime-clavulanic acid discs versus cefotaxime or ceftazidime discs alone was considered as a sign of ESBL-production by *E. coli* isolates. *E. coli* ATCC® 25,922 strain was also included in this test for quality control [22].

### Detection of major ESBL genes in *E. coli* isolates

To detect major ESBL genes in *E. coli* isolates, the DNA of *E. coli* isolates was extracted by boiling methods. Detection of *bla*_CTX-M_, *bla*_SHV_, and *bla*_TEM_ genes was performed using PCR and agar gel electrophoresis techniques, as previously described [23].

### Detection of class 1 and class 2 integrons in *E. coli* isolates

A duplex PCR was conducted to amplify the 483-bp fragment of class 1 integron-integrase (*intI1*) gene and the 789-bp fragment of class 2 integron-integrase (*intI2*) gene, according to a protocol by Shaheen et al. [24] with minor modifications in the annealing temperature, which was adjusted at 64 °C. Electrophoresis of the PCR products was completed on 1% agarose (Parstous, Iran) gel containing a safe stain (YTA, Iran). Next, the amplicons were visualized under an ultraviolet-transilluminator (UVitec, UK).

### Determination of antibiotic resistance patterns of *E. coli* isolates

The Kirby-Bauer disk diffusion test was applied for determination of antibiotic resistance patterns of *E. coli* isolates against cephalexin (30 μg), cefoxitin (30 μg), ceftazidime (30 μg), cefotaxime (30 μg), cefepime (30 μg), aztreonam (30 μg), amikacin (30 μg), streptomycin (10 μg), norfloxacin (10 μg), nalidixic acid (30 μg), chloramphenicol (30 μg), and tetracycline (30 μg) (Padtan Teb, Iran). Interpretation of the results was conducted according to the guidelines of Clinical and Laboratory Standards Institute (CLSI) [21]. The strain of *E. coli* ATCC® 25,922 was included in this study as a quality control [21].

### Statistical analysis

Analysis of data was performed with SPSS software (version 16.0, SPSS Inc., Chicago, IL, USA). The Chi-square test was used to compare the results in different studied groups. A value of *P* ≤ 0.05 was considered significant.

## Data Availability

The data are available from the corresponding author on reasonable request.

## Abbreviations

MDR: Multi-drug resistance
ESBL: Extended spectrum beta-lactamase
*bla*: Beta-lactamase
*E. coli*: *Escherichia coli*
ESCRE: Extended-spectrum cephalosporin-resistant *Enterobacteriaceae*
*dfr*: Dihydrofolate reductase
*aad*: Aminoglycoside adenyltransferase
PFGE: Pulsed-field gel electrophoresis
MLST: Multi-locus sequence typing
ERIC: Enterobacterial repetitive intergenic consensus
RAPD: Randomly amplified polymorphic DNA
PCR: Polymerase chain reaction
CLSI: Clinical and laboratory standards institute
ATCC®: American type culture collection
*intI*: Integron-integrase
SPSS: Statistical product and service solutions
